# Illustration of children's blood pressure in diabetes mellitus type 1 in Indonesia

**DOI:** 10.1186/1687-9856-2013-S1-P12

**Published:** 2013-10-03

**Authors:** Indra Himawan, GM Annang, Aman B Pulungan

**Affiliations:** 1 Department of children health of medical faculty of Lambung Mangkurat University, Banjarmasin, Indonesia; 2 Department of children health of medical faculty of Sebelas Maret University, Solo, Indonesia; 3 Department of children health, medical faculty of University of Indonesia Jakarta – Cipto Mangunkusumo Hospital, Jakarta, Indonesia

## Background

One of the long-term complications of diabetes mellitus type 1 (DM type 1) in children is diabetic nephropathy. Diabetes longstanding suffered and pubertal status are the influencing factors which make the complications come up to the surface. Diabetic nephropathy case will be accompanied by increasing of blood pressure symptoms.

## Objective

To detect the illustration of childrens blood pressure in DM type 1 base on their age, sex, longstanding suffered, and the latest level of HbA1c.

## Methods

A retrospective observational studies on national registered of DM type 1 at pediatric endocrinology chapter to 2010. Age, sex, longstanding suffered, the latest level of HbA1c, systolic blood pressure (SBP) and dyastolic (DBP) are variables which has been researched.

## Result

Starting at 177 children suffered by DM type 1, 118 (66.7%) are girls and 59 (33.3%) are boys. The average age when they got diagnose for the first time is 11,8 years old. Only one girl (0.6%) who suffered DM type 1 more than 5 years, has SBP and DBP > percentile 95, There is no any significant correlation with the others.

## Conclusion

Most of the childrens blood pressure in DM type 1 are still normal. In this research, a girl who being suffered in DM type 1 with hypertension (> 95th percentile) has been being suffered more than 5 years. It needs doing blood pressure measurement regularly, for screening the diabetic nephropathy, if s microalbumin examination has not been done yet.

